# Genetic modifiers of penetrance to liver endpoints in *HFE* hemochromatosis: Associations in a large community cohort

**DOI:** 10.1002/hep.32575

**Published:** 2022-06-17

**Authors:** Luke C. Pilling, Janice L. Atkins, David Melzer

**Affiliations:** ^1^ Epidemiology and Public Health Group University of Exeter Exeter UK

## Abstract

**Background:**

The iron overload condition hereditary hemochromatosis (HH) can cause liver cirrhosis and cancer, diabetes, and arthritis. Males homozygous for the p.C282Y missense mutation in the Homeostatin Iron Regulator (*HFE*) gene have greatest risk; yet, only a minority develop these conditions. We aimed to determine whether common genetic variants influencing iron levels or liver disease risk in the general population also modify clinical penetrance in *HFE* p.C282Y and p.H63D carriers.

**Methods:**

We studied 1294 male and 1596 female UK Biobank *HFE* p.C282Y homozygous participants of European ancestry with medical records up to 14 years after baseline assessment. Polygenic scores quantified genetic effects of blood iron biomarkers and relevant diseases (identified in the general population). Analyses were also performed in other *HFE* p.C282Y/p.H63D genotype groups.

**Results:**

In male p.C282Y homozygotes, a higher iron polygenic score increased the risk of liver fibrosis or cirrhosis diagnoses (odds ratio for the top 20% of iron polygenic score vs. the bottom 20% = 4.90: 95% confidence intervals, 1.63–14.73; *p* = 0.005), liver cancer, and osteoarthritis but not diabetes. A liver cirrhosis polygenic score was associated with liver cancer diagnoses. In female p.C282Y homozygotes, the osteoarthritis polygenic score was associated with increased osteoarthritis diagnoses and type‐2 diabetes polygenic score with diabetes. However, the iron polygenic score was not robustly associated with diagnoses in p.C282Y female homozygotes or in other p.C282Y/p.H63D genotypes.

**Conclusions:**

*HFE* p.C282Y homozygote penetrance to clinical disease in a large community cohort was partly explained by common genetic variants that influence iron and risks of related diagnoses in the general population, including polygenic scores in HH screening and diagnosis, may help in estimating prognosis and treatment planning.

## Graphical abstract

Within males homozygous for the missense mutation p.C282Y in the Homeostatic Iron Regulator (*HFE*) gene those carrying greater number of other iron‐increasing genetic variants had significantly raised likelihood of diagnosis of liver endpoints.

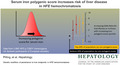

**Abbreviations**
CI: confidence interval
GP: general practice
GWAS: genome‐wide association studies
HEIRS: Hemochromatosis and Iron Overload Screening
HES: hospital episode statistics
HH: hereditary hemochromatosis
ICD‐10: International Classification of Diseases 10th revision
MR: Mendelian randomization
OR: odds ratio
SNP: single‐nucleotide polymorphism
T2D: type‐2 diabetes
*TF*
: transferrin
TIBC: total iron‐binding capacity
*TMPRSS6*
: transmembrane serine protease 6
UKB: UK Biobank

## INTRODUCTION

Hereditary hemochromatosis (HH) is a genetic condition associated with iron overload, which, in European ancestry groups, is predominantly caused by the missense mutation p.C282Y in the Homeostatic Iron Regulator (*HFE*) gene (homozygote mutation in >95% of cases), with some additional diagnoses in p.H63D homozygotes.^[^ ^1^ ^]^ The p.C282Y mutation leads to reduced plasma hepcidin levels, raised ferritin and transferrin (*TF*) saturation levels, and a gradual accumulation of systemic iron in adults.^[^ ^2^ ^]^ Clinical presentations of the condition include fatigue, arthropathy (osteoarthritis is a common and sometimes severe symptom^[^ ^3^ ^]^), diabetes, liver disease, and hormone dysregulation, and the disease can progress to liver cirrhosis, liver cancer, and cardiomyopathy.^[^ ^4^ ^]^ However, penetrance to clinical symptoms or disease is limited: in the UK Biobank (UKB) study—the largest community study thus far if *HFE* p.C282Y homozygotes (*n* = 2890)—we estimated that only 25.3% of p.C282Y homozygous male participants and 12.5% of homozygous female participants were diagnosed with hemochromatosis by the age of 65 years.^[^ ^5^ ^]^ These estimates were similar to a 2015 study across seven American medical systems (eMERGE^[^ ^6^ ^]^: *n* = 106 homozygotes) that reported that 24.4% of male and 14.0% of female p.C282Y homozygotes were diagnosed with hemochromatosis (mean age 66.4 ± 15.8 years), with Kaplan Myer survival curves suggesting 50% of the homozygote men and 25% of homozygote women were eventually diagnosed with hemochromatosis by the age of 90 years.

This limited clinical penetrance may be explained in part by environmental factors,^[^ ^7^ ^]^ including high alcohol consumption and hepatitis C virus infection for liver fibrosis or cirrhosis, but there is also evidence for genetic factors being involved.^[^ ^8^ ^]^ For example, in a genome‐wide association studies (GWAS) in 474 unrelated p.C282Y homozygotes, single‐nucleotide polymorphism (SNP) rs3811647 in the *TF* gene was associated with serum iron but not clinical phenotypes.^[^ ^9^ ^]^ It explained 7.7% of the variance of serum *TF* concentration and 4.7% of the variance of serum iron levels. We previously reported preliminary evidence that common variants affecting iron levels may interact with p.C282Y genotype to increase risk of disease,^[^ ^10^ ^]^ but more evidence is required to understand the modifying effects of common variants on p.C282Y penetrance. Effects in *HFE* genotype groups other than p.C282Y homozygotes are disputed, with the recent recommendations for hemochromatosis classification by the BIOIRON society^[^ ^11^ ^]^ suggesting that other genotypes (including p.C282Y/p.H63D compound heterozygotes, p.C282Y heterozygotes, and p.H63D status) require additional evidence for hemochromatosis diagnosis because of minimal or no clinical penetrance.^[^ ^10^ ^]^ Factors modifying penetrance in these groups remain to be fully determined.

HH appears to meet several criteria for genetic screening,^[^ ^12^ ^]^ but the low clinical penetrance in community p.C282Y homozygotes was a major factor in limiting screening to close relatives. A better understanding of the limited penetrance might improve prediction of prognosis and might allow more targeted screening for those at risk of disease. We therefore aimed to identify whether common variants linked to variation in iron measures are associated with a clinical diagnosis of HH and related outcomes (especially liver fibrosis or cirrhosis) within *HFE* p.C282Y homozygotes and separately in other *HFE* genotype groups. We used the UKB, a cohort of over 500,000 community volunteers receiving routine clinical care; the UKB consent procedure explicitly involved no personal feedback of genetic findings.

## PARTICIPANTS AND METHODS

The UKB study includes data on 502,634 volunteers aged 40–70 years at baseline study invitation. Recruitment was through postal invitation to people registered with the UK National Health Service living within 40 km of 22 assessment centers in England, Scotland, and Wales. Participants consented to genotyping and for data linkage for follow‐up by hospital admission medical records (hospital episode statistics [HES]), cancer registry, primary care (general practice [GP]), and death certificates. UKB volunteers tended to be healthier at baseline than the general UK population.^[^ ^13^ ^]^

Data are available to any bone fide researcher following an application to the UKB (www.ukbiobank.ac.uk/register‐apply). The North West Multi‐Centre Research Ethics Committee approved the collection and use of UKB data for studies such as these (Research Ethics Committee reference11/NW/0382) thus the requirement for informed consent was waived by the review committee. The study protocol conformed to the ethical guidelines of the 1975 Declaration of Helsinki. Access to the UKB was granted under application number 14631.

### Disease ascertainment

Disease ascertainment was by subject responses to questionnaire items on doctor‐diagnosed diseases at baseline (2006–2010, which were verified by a trained nurse), combined with International Classification of Diseases 10th revision (ICD‐10)–coded hospital inpatient records, cancer registry data, and read codes from primary care data that were available for approximately 45% of the participants. The censoring dates for the three sources of electronic medical records were up to September 2021 for HES for England (July 2021 for Scotland and February 2018 for Wales), July 2019 for cancer register for England and Wales (October 2015 for Scotland), and August 2017 for primary care for Wales (March 2017 for Scotland, May 2017 for England Vision supplier, and August 2016 for England TPP supplier). Diagnoses ascertained were hemochromatosis (ICD‐10 code E83.1), liver fibrosis or cirrhosis (ICD‐10 codes K74*), liver cancer (ICD‐10 code C22*), diabetes mellitus type 2 (ICD10 code E11*), and osteoarthritis (ICD‐10 codes M15.0, M15.1, M15.2, M15.9, M16.0, M16.1, M17.0, M17.1, M18.0, M18.1, and M19.0). Corresponding primary care diagnosis codes were identified using the UKB “Clinical coding classification systems and maps” resource to map ICD‐10 codes to Read2/CTV3 (https://biobank.ctsu.ox.ac.uk/crystal/refer.cgi?id=592).

### Genotyping in UKB

Participants were genotyped using two almost identical (>95% shared variants, *n* = 805,426 total) microarray platforms: the Affymetrix Axiom UKB array (in 438,427 participants) and the Affymetrix UKBiLEVE array (in 49,950 participants). The central UKB team performed genotype imputation in 487,442 participants, increasing the number of genetic variants to ~96 million.^[^ ^14^ ^]^ Because *HFE* p.C282Y is largely restricted to Europeans, we analyzed 451,427 (93%) participants who self‐reported as “White European” and were confirmed as being of genetically European ancestry (described^[^ ^15^ ^]^). A total of 445,521 participants (98.7% of 451,427) had *HFE* p.C282Y (rs1800562) imputed with 100% confidence, and 5723 were recoded (i.e., estimated genotype dose between 0 and 0.25 set to 0, values between 0.75 and 1.25 set to 1, and, finally, between 1.75 and 2 set to 2); 183 participants (0.04%) were excluded because of imprecise imputation, yielding 451,243 participants in analyses. *HFE* p.H63D (rs1799945) was directly genotyped on the microarray.

### Polygenic scores for iron status biomarkers

We created polygenic scores for four iron status biomarkers using 128 non‐*HFE* variants (p.C282Y/p.H63D variants excluded) identified in a GWAS of 257,953 individuals.^[^ ^16^ ^]^ We used 20 variants associated with iron itself, 64 associated with ferritin, 19 with *TF* saturation, and 41 with total iron‐binding capacity (TIBC; Table S1). We excluded a small number of variants identified in the original GWAS if they were not present in the UKB imputed data (v3), if the minor allele frequency was <0.1%, if there was significant deviation from Hardy‐Weinberg equilibrium (*p* < 5 × 10^−8^), or if the imputation quality (INFO score) was below 80% (see Table S2 for details). For each participant, the number of trait‐raising alleles was counted, weighted by the effect size reported in the published GWAS^[^ ^16^ ^]^ (effects and effect alleles reported in Table S2; excluded SNPs with criteria are listed in Table S2).

### Polygenic risk scores for hemochromatosis‐associated comorbidities

Genetic variants associated with liver cirrhosis, osteoarthritis, and type‐2 diabetes (T2D) in general population studies (i.e., not specific to hemochromatosis) were identified from published GWAS,^[^ ^17^, ^18^, ^19^ ^]^ and for each participant, the number of trait‐raising alleles was counted and weighted by the effect size reported in the published GWAS. A small number of variants identified in the original GWAS were excluded if the SNP was +/−250 kb of p.C282Y, they were not present in the UKB imputed data (v3), if the minor allele frequency was <0.1%, if there was significant deviation from the Hardy‐Weinberg equilibrium (*p* < 5 × 10^−8^), or if the imputation quality (INFO score) was below 80%.

### Missing data

We excluded participants without imputed genotype data (*n* = 15,233/502,642, 3.03%), those with imprecise imputation for p.C282Y (*n* = 183/487,409, 0.037%), and those who had withdrawn from the study at the time of analysis (December 2021). Less than 0.5% of participants had no answers to questions on self‐reported diseases. Given the low level of missing data, we excluded participants with missing data from individual analysis, as needed.

### Statistical analysis

Polygenic scores represent life‐long predisposition to higher/lower levels of iron status biomarkers; we therefore used logistic regression models to test the hypothesis that “polygenic score for trait X is associated with ever bring diagnosed with outcome Y,” adjusted for age at end of medical records follow‐up, assessment center, and genetic principal components of ancestry 1–10.

To adjust for multiple statistical testing and reduce the false discovery rate, we used the Benjamini‐Hochberg method to identify *p* values < 0.05 after multiple testing correction. These are indicated on the figures and in the text.

We applied two‐sample Mendelian randomization (MR) methods to test the robustness of the associations seen using the two‐stage least squares approach (i.e., the one‐sample approach testing associations among iron polygenic score outcomes within UKB). We used the R package RadialMR to test for significant pleiotropy (using the MR Egger approach^[^ ^20^ ^]^) and significant outliers or heterogeneity in the variant effects (RadialMR approach).^[^ ^21^ ^]^

## RESULTS

In 2890 *HFE* p.C282Y homozygotes in UKB participants of European ancestry, we identified 771 (26.7%) with a hemochromatosis diagnosis at the end of available electronic medical record data (HES up to September 2021 or GP data up to September 2017; GP data were available in 45% of cohort). Diagnosis was more common in male participants (33.2% of 1294 male participants vs. 21.4% of 1596 female participants), and mean age at diagnosis was 61.5 years (60.1 in male participants). See Table 1. In non‐p.C282Y homozygotes, diagnosis was substantially less common (703 diagnoses in 448,441 participants), as expected (see Table S3 for details including number of comorbidity diagnoses in other *HFE* genotype groups).

**TABLE 1:** Characteristics of the UK Biobank *HFE* p.C282Y homozygote participants of European ancestry, stratified by sex

	All		Male participants		Female participants	
	N	%	N	% (M)	N	% (F)
*HFE* p.C282Y homozygotes	2890		1294		1596	
Diagnosis^a^:						
Hemochromatosis	771	26.7	430	33.2	341	21.4
Liver fibrosis or cirrhosis	78	2.7	60	4.6	18	1.1
Liver cancer	30	1.0	27	2.1	3	0.2
Osteoarthritis	721	24.9	326	25.2	395	24.7
Type‐2 diabetes	266	9.2	163	12.6	103	6.5
	**Mean**	**SD**	**Mean**	**SD**	**Mean**	**SD**
Age (end follow‐up or death)	69.6	8.0	69.3	8.1	69.8	8.0
Age at hemochromatosis diagnosis	61.5	9.3	60.1	9.7	63.2	8.5

Abbreviations: % (F), percentage of female participants; % (M), percentage of male participants; GP, general practice; HES, hospital episode statistics.

^a^ Diagnosis ever recorded in data from baseline self‐report, HES up to September 2021, cancer registry up to July 2019, or GP data up to September 2017 (GP data available in 45% of cohort).

### Polygenic risk score of common iron‐increasing genetic variants affects penetrance in *HFE* p.C282Y homozygous participants

**FIGURE 1:**
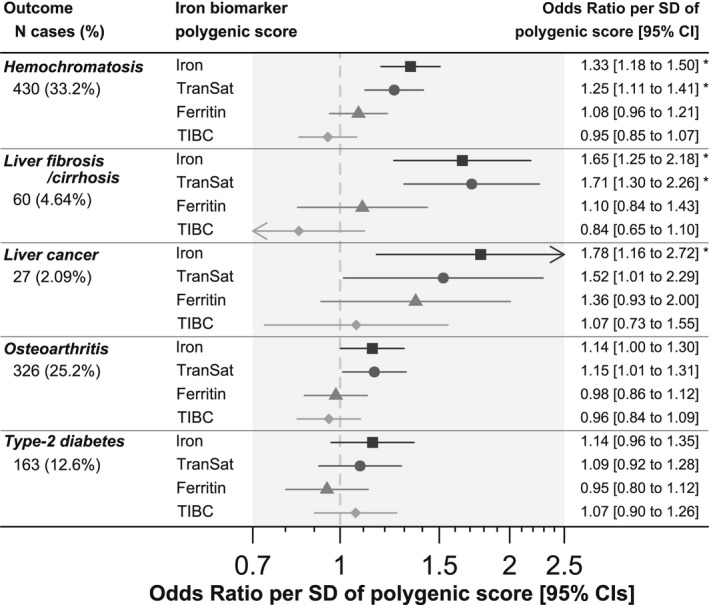
Linear associations among four iron status biomarker polygenic scores and hereditary hemochromatosis comorbidities in Homeostatin Iron Regulator *(HFE)* p.C282Y homozygous male participants. Results are from logistic regression models adjusted for age, assessment center, and principal components of ancestry 1 to 10. Percentage (%) of 1294 *HFE* p.C282Y homozygous male participants of European genetic ancestry who ever received a diagnosis in the available data (up to September 2021). See Table S4 for details, including associations in women. CI, confidence interval; TIBC, total iron‐binding capacity; TranSat, transferrin saturation. *p < 0.05 (false discovery rate adjusted using the Benjamini‐Hochberg method). Arrows indicate where the CIs go beyond the x axis limits.

We tested associations among polygenic scores for four blood iron status biomarkers^[^ ^16^ ^]^ and hemochromatosis‐associated comorbidities in male *HFE* p.C282Y homozygotes (Figure 1).

Iron polygenic score was associated with increases in likelihood of ever being diagnosed with related diseases, especially liver fibrosis or cirrhosis (odds ratio [OR] per SD increase in iron polygenic score, 1.65: 95% confidence intervals [CIs], 1.25–2.18; *p* = 5 × 10^−4^) and liver cancer (OR, 1.69: 95% CI, 1.01–2.81; *p* = 0.04) in logistic regression models adjusted for age, assessment center, and principal components of ancestry 1–10 (Table S4) after adjustment for multiple testing. Iron polygenic score was also nominally associated with increased likelihood of osteoarthritis (OR, 1.14: 95% CI, 1.00–1.30; *p* = 0.046), but trends with T2D (OR, 1.14: 95% CI, 0.96–1.35; *p* = 0.12) did not reach significance. Iron polygenic score was also significantly associated with greater likelihood of ever receiving a hemochromatosis diagnosis (OR, 1.33: 95% CI, 1.18–1.50; *p* = 3 × 10^−6^). In female *HFE* p.C282Y homozygotes, iron polygenic score increased likelihood of hemochromatosis diagnosis (OR, 1.32: 95% CI, 1.17–1.49; *p* = 1 × 10^−5^) but was not associated with any comorbidities tested (*p* > 0.05; Table S4).

We also created a polygenic score for *TF* saturation using 19 genetic variants, and results were highly similar to the iron polygenic score results reported above (Figure 1; Table S4); however, polygenic scores for ferritin and TIBC were not associated with diagnosis of hemochromatosis or any comorbidities (*p* > 0.05; Figure 1; Table S4).

**FIGURE 2:**
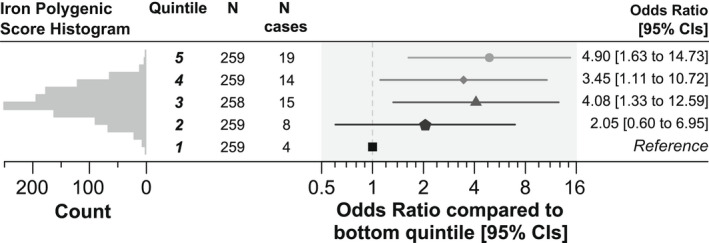
Iron polygenic score association with diagnosis of liver fibrosis or cirrhosis in Homeostatin Iron Regulator *(HFE)* p.C282Y homozygous male participants. Iron polygenic score is stratified into five equally sized groups (quintiles). Results are from logistic regression models adjusted for age, assessment center, and principal components of ancestry 1 to 10. *N* = *HFE* p.C282Y homozygous male participants of European genetic ancestry in quintile. *N* cases = participants in quintile who ever received a diagnosis of liver fibrosis or cirrhosis in the available data (up to September 2021). Iron polygenic score is the score for total iron levels. See Table S5 for details including polygenic score cut points. CI, confidence interval.

To explore the association between iron polygenic score and diagnosis of liver fibrosis or cirrhosis further, we stratified the 1294 male *HFE* p.C282Y homozygotes into five equally sized groups (quintiles) based on their iron polygenic score. Those into the top 20% of iron polygenic score (*n* = 259) had substantially higher likelihood of being diagnosed with liver fibrosis or cirrhosis (*n* = 19) compared with those in the bottom 20% of iron polygenic score (*n* = 4 diagnoses in 259 participants) (OR, 4.90: 95% CI, 1.63–14.73; *p* = 0.005) (Figure 2; Table S5).

### Iron‐increasing genetic variants and hemochromatosis comorbidities in other *HFE* genotype groups

**FIGURE 3:**
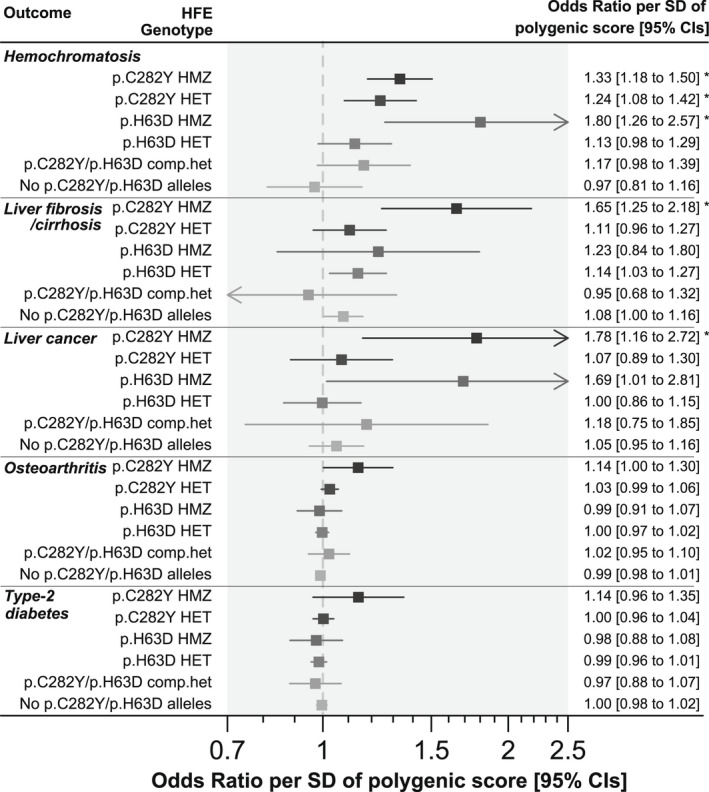
Linear increases in iron polygenic scores and hereditary hemochromatosis comorbidities in other Homeostatin Iron Regulator *(HFE)* genotype groups. Analysis performed in UK Biobank male participants of European ancestry. Results are from logistic regression models adjusted for age, assessment center, and principal components of ancestry 1 to 10. Percentage (%) of *HFE* genotype group who ever received a diagnosis in the available data (up to September 2021). See Table S4 for details, including associations in women. CI, confidence interval. HMZ = homozygotes. HET = heterozygotes. comp.het = compound heterozygotes. *p < 0.05 (false discovery rate adjusted using the Benjamini‐Hochberg method). Arrows indicate where the CIs go beyond the x axis limits.

Iron polygenic score was not associated with diagnosis of hemochromatosis comorbidities in male p.C282Y heterozygotes or p.C282Y/p.H63D compound heterozygotes (Figure 3; Table S4). Iron polygenic score was nominally associated with increased likelihood of liver fibrosis/cirrhosis in p.H63D heterozygotes (OR per SD of polygenic score, 1.14: 95% CI, 1.03–1.27; *p* = 0.02), and separately with liver cancer in p.H63D homozygotes (OR, 1.69: 95% CI, 1.01–2.81; *p* = 0.04), though neither were significant after adjustment for multiple statistical testing. Iron polygenic score significantly increased likelihood of diagnosis of hemochromatosis itself in p.C282Y heterozygotes (OR, 1.24: 95% CI 1.08–1.42; *p* = 0.002) and p.H63D homozygotes (OR, 1.80: 95% CI, 1.26–2.57; *p* = 0.001) but not p.C282Y/p.H63D compound heterozygotes or p.H63D heterozygotes (*p* > 0.05) (Figure 3; Table S4).

A polygenic score for *TF* saturation showed similar patterns of association with outcomes to the iron polygenic score, especially with increasing likelihood of hemochromatosis diagnosis in p.H63D homozygotes (OR per SD of polygenic score, 2.15: 95% CI 1.49–3.09; see Table S4 and Figure S1). A polygenic score for ferritin was not associated with hemochromatosis‐associated outcomes in any of the *HFE* genotype groups tested (see Table S4 and Figure S2). TIBC polygenic score was nominally associated with increased likelihood of hemochromatosis diagnosis in p.H63D homozygotes only (OR per SD increase in polygenic score, 1.51: 95% CI, 1.05–2.18; *p* = 0.03; see Table S4 and Figure S3).

### Comorbidity polygenic score associations in *HFE* p.C282Y homozygotes

**FIGURE 4:**
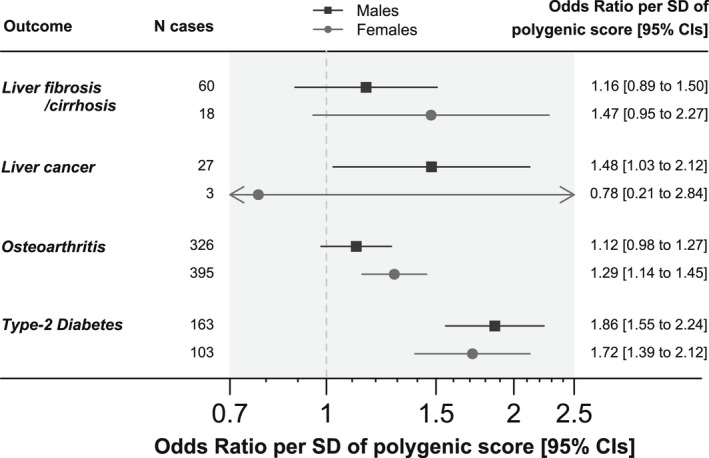
Linear increases in polygenic scores for hereditary hemochromatosis comorbidities affect likelihood of corresponding diagnosis in Homeostatin Iron Regulator *(HFE)* p.C282Y homozygotes. Results are from logistic regression models adjusted for age, assessment center, and principal components of ancestry 1 to 10. Liver cirrhosis polygenic score was tested against diagnoses of liver fibrosis or cirrhosis and separately against diagnoses of liver cancer. Osteoarthritis polygenic score was tested against diagnoses of osteoarthritis and type‐2 diabetes (T2D) and polygenic score against T2D. See Table S6 for details. CI, confidence interval. Arrows indicate where the CIs go beyond the x axis limits.

Within *HFE* p.C282Y homozygous male participants, a polygenic score for liver cirrhosis was nominally associated with increased risk of liver cancer (OR, 1.48: 95% CI, 1.03–2.12; *p* = 0.04) (Figure 4; Table S6). The association was not significantly different to that in the *HFE* wild‐type group (no p.C282Y or p.H63D genotypes) when an interaction term was included between cirrhosis polygenic score and *HFE* genotype (*p* > 0.05). A polygenic score for osteoarthritis was significantly associated with diagnosis of osteoarthritis in *HFE* p.C282Y homozygous female participants (OR, 1.29: 95% CI, 1.14–1.45; *p* = 4 × 10^−5^) but not male participants (OR, 1.12: 95% CI, 0.98–1.27; *p* = 0.1). The association in p.C282Y homozygous female participants was significantly greater than that in *HFE* wild‐type participants (interaction *p* = 0.012). A polygenic score for T2D was significantly associated with increased likelihood of T2D diagnosis in both p.C282Y homozygous male participants (OR, 1.86: 95% CI, 1.55–2.24; *p* = 2 × 10^−11^) and female participants (OR, 1.72: 95% CI, 1.39–2.12; *p* = 6 × 10^−7^), though in both cases, the association did not significantly differ from that seen in *HFE* wild‐type genotype participants (interaction *p* > 0.05). The liver cirrhosis polygenic score was not associated with diagnosis of liver cirrhosis in p.C282Y homozygotes (*p* > 0.05), though the association was significant in the larger *HFE* wild‐type group (OR_males_, 1.20: 95% CI, 1.16–1.23; *p* = 6 × 10^−38^).

### Sensitivity analysis

The primary analysis in UKB included all male participants of European ancestry who were homozygous for *HFE* p.C282Y (*n* = 1294): in sensitivity analysis, we identified 13 pairs of participants related to the third degree or closer (using KING Kinship based INference for Gwas analysis^[^ ^22^ ^]^). We randomly excluded one of each pair of related participants and repeated the primary analysis of iron polygenic score associations with HH comorbidities in unrelated *HFE* p.C282Y homozygous European male participants. The associations between iron polygenic score and outcomes remained consistent, suggesting the result was not biased by inclusion of related participants (Table S7).

We repeated the primary analysis of iron polygenic score associations with diagnosis of liver fibrosis/cirrhosis and separately liver cancer using two‐sample MR methods. We found no evidence for pleiotropy (MR Egger intercept *p* values > 0.05) or bias because of outliers (see Table S8 for results), suggesting the primary analysis results presented are robust.

## DISCUSSION

In population studies, *HFE* p.C282Y homozygosity is associated with high biochemical penetrance (to raised iron measures) but low penetrance to hemochromatosis‐related clinical diagnoses.^[^ ^7^, ^23^ ^]^ Many genetic variants are known to influence iron measures in the general population and risk of liver disease, arthritis, or diabetes; although most individual effects are small, the cumulative expected effects of risk alleles can be computed into polygenetic scores for each study participant. We therefore tested whether these polygenic scores could explain some of the variance in clinical penetrance with the high‐risk *HFE* p.C282Y homozygous group. We found that carrying a greater number of common genetic variants, increasing serum iron and *TF* saturation levels, increased the incidence of HH‐associated diseases in 2890 *HFE* p.C282Y homozygotes and other *HFE* genotypes in the UKB, the largest community study thus far of p.C282Y homozygotes (*n* = 2890). We also found that p.C282Y homozygotes with high polygenic risk for liver cirrhosis, osteoarthritis, or diabetes, were more likely to develop those specific comorbidities. Our results support the conclusion that the variable clinical penetrance of HH seen in *HFE* p.C282Y homozygotes is partly attributable to the burden of polygenic risk for higher iron and higher risk of comorbidities.

A recent GWAS meta‐analysis of iron status biomarkers (irrespective of *HFE* genotype) in 257,953 individuals identified 127 loci.^[^ ^16^ ^]^ This included loci with well‐established roles in iron homeostasis and metabolism, such as *TF*, ferroportin‐1, and transmembrane serine protease 6 (*TMPRSS6*). The effect sizes for non‐*HFE* variants are modest. Several previous candidate gene studies have investigated the role of modifying genetic variants amongst iron metabolism genes in hemochromatosis (such as^[^ ^8^, ^24^ ^]^), yet sample size was a limitation. In UKB we were able to extend these studies and use polygenic scores and MR methods to robustly model the cumulative risk of many small‐effect genetic variants.

Higher polygenic score for serum iron and for *TF* saturation increased risk of liver disease, especially liver fibrosis or cirrhosis and liver cancer. Progressive increases in serum levels are markers of increased iron absorption and are amongst the earliest signs of hemochromatosis.^[^ ^25^ ^]^ Progressively increasing serum ferritin (hyperferritinemia) is also characteristic of hemochromatosis,^[^ ^25^ ^]^ reflecting increasing iron storage; although ferritin levels are raised in several conditions including acute inflammation, which may have resulted in the weaker association trends between a higher polygenic score for ferritin (or TIBC) and an increase in risk of liver disease or comorbidities. Raised serum iron biomarkers are reported in other *HFE* genotype groups, especially in p.C282Y/p.H63D compound heterozygotes compared with noncarriers;^[^ ^26^ ^]^ however, evidence for the impact on clinical diagnosis and morbidity is variable.^[^ ^27^ ^]^ We found that genetic predisposition to higher serum iron did not increase risk of hemochromatosis‐associated comorbidity in these other *HFE* genotype groups.

Hepcidin is the key hormone regulating iron absorption by binding to ferroportin, limiting the release on iron into the blood.^[^ ^28^ ^]^ *HFE* mutations result in reduced hepcidin expression in the liver and thus increased iron absorption. Included in the iron polygenic score are variants mapped to genes known to regulate the hepcidin cascade, such as *TMPRSS6*, a liver‐specific transmembrane protein that increases hepcidin production.^[^ ^29^ ^]^ That no variants were identified in the hepcidin gene itself (*HAMP*, Hepcidin Antimicrobial Peptide) supports the hypothesis that for most patients it is the cascade events upstream of hepcidin (starting with *HFE*) that leads to hepcidin dysregulation and iron overload.^[^ ^30^ ^]^ We saw no significant outlier variants in the MR analysis, confirming that the polygenic score results were not driven by a small number of effects in key genes such as *TMPRSS6*, but rather are the average effect of all iron‐increasing variants.

Preventative treatment such as phlebotomy is safe and effective, and therefore, efforts to diagnose the high‐risk p.C282Y homozygote group earlier are needed to reduce morbidity.^[^ ^4^ ^]^ In UKB, only 33% of male and 21% of female p.C282Y homozygotes were diagnosed with hemochromatosis by the end of available medical record data (mean age 69 and 70 years, respectively). Higher polygenic score for serum iron was associated with increased likelihood of both excess morbidity and hemochromatosis diagnosis in p.C282Y homozygotes. In study participants with the other *HFE* variants studied (including p.C282Y/p.H63D compound heterozygotes, p.C282Y heterozygotes, and p.H63D genotypes separately), the iron polygenic risk score was not associated with excess morbidity, consistent with the lack of statistical evidence for overall excess morbidity in these latter genotype groups;^[^ ^10^, ^11^ ^]^ that is, genotypes other than p.C282Y homozygotes have no apparent clinical consequences (even in those with higher iron polygenic score for the studied outcomes), and therefore, recent guidelines indicate that diagnosis of hemochromatosis is not needed in the absence of additional factors. Nevertheless, the iron polygenic score was associated with being diagnosed as having hemochromatosis in non‐p.C282Y homozygotes, perhaps because of clinicians mistaking higher iron blood measures with a need for hemochromatosis diagnosis, although more work is needed to confirm this apparent misdiagnosis.

Others have suggested a multifactorial model of HH characterized principally by variants in *HFE* with modifying effects of genetic and environmental factors that are yet to be fully determined.^[^ ^8^ ^]^ Environmental factors such as alcohol consumption and hepatitis C virus infection appear to increase susceptibility to iron overload, with roles for insulin resistance, fatty acid accumulation, and ineffective erythropoiesis.^[^ ^31^ ^]^ Protective factors are also reported, including a correct diet and positive attitude to blood donations.^[^ ^32^ ^]^ Incomplete clinical penetrance is partly explained by these factors, and yet, there is also incomplete biochemical penetrance within *HFE* genotype groups: the Hemochromatosis and Iron Overload Screening (HEIRS) study reported that although in undiagnosed male p.C282Y homozygotes mean *TF* saturation was 76% compared with 32% in male participants without *HFE* mutations, there were still 16% of p.C282Y homozygotes men with *TF* saturation below 50%.^[^ ^26^ ^]^ The polygenic score for *TF* saturation is reported to explain 11% of variance in *TF* saturation (although this polygenic score included *HFE* variants) in 56,664 participants from the Trøndelag Health (HUNT) study, strongly supporting the hypothesis that common non‐*HFE* variants modify biochemical penetrance, and our results support that this impacts penetrance to clinical disease, especially in the liver.

Limitations of this analysis include that UKB volunteers tended to be healthier than the general population^[^ ^13^ ^]^ at baseline, although this effect may have diminished during the long observed electronic medical records follow‐up of over 14 years. Though hospital inpatient diagnoses were available for all participants, primary care data were only available in approximately 45% of the cohort. For example, we identified 617 hemochromatosis diagnoses in the 45% subset using hospital inpatient data, which increased by 28% to 791 when also including diagnoses present in the primary care data. Therefore, estimates of penetrance to disease may be underestimated because 55% of UKB participants were missing primary care data. It is possible that response rates to UKB may have been affected by *HFE* mutation status or associated morbidity, but as previously reported, the overall prevalence of p.C282Y homozygosity (one in 156) was very similar to previous reports for groups of British or Irish descent,^[^ ^10^ ^]^ and the p.C282Y variant was in Hardy‐Weinberg equilibrium (*p* > 0.05) in UKB, implying that the observed genotypes are present in the expected proportions, with no sign of differential loss or excess of p.C282Y homozygotes. The UKB sample included a wide range of exposures and socioeconomically diverse groups,^[^ ^13^ ^]^ and prospective analyses are less affected by sample response patterns at baseline. These factors suggest that our results are robust and likely to be applicable to the United Kingdom and other European descent populations. Though iron status biomarkers were not measured in UKB, biochemical penetrance of *HFE* mutations is well documented by the HEIRS study amongst others,^[^ ^26^ ^]^ and the effect of identified variants is reliably reported by the HUNT meta‐analysis.^[^ ^16^ ^]^ We did not find a significant association between a polygenic score for liver cirrhosis and diagnosis of fibrosis or cirrhosis, which may be due to the limited contribution of hemochromatosis to liver endpoints in the general population.

Strengths of our analysis include that UKB is the largest community genotyped study of p.C282Y homozygotes (nearly 10 times bigger than HEIRS^[^ ^26^ ^]^). We had good ascertainment of clinical diagnoses through primary care electronic medical records and hospital admission data, though as noted above, penetrance may be underestimated as primary care data are only available for ~45% of participants. Very few p.C282Y homozygotes were diagnosed with hemochromatosis at baseline (12% of male participants),^[^ ^10^ ^]^ and participants consented to not be told about UKB‐ascertained genotypes, so results are similar to what might be expected from a community screening.

The UKB sample included some sets of related individuals, as assessed through genome‐wide variant similarity (KING kinship coefficient). In sensitivity analysis excluding one of each pair of participants related to the third degree or closer, the results were unaffected. Unfortunately, there are no data in UKB on whether each related or unrelated UKB participant was from a family with a strong history of hemochromatosis diagnoses or not. Current screening focuses on families, that is, first‐degree relatives of p.C282Y homozygotes, though this only identifies a minority of homozygotes; an Australian study estimated that only 2.9% of male homozygotes and 2.0% of female homozygotes were identified in family screening.^[^ ^33^ ^]^ Our results show that family relatedness did not affect associations, supporting calls for family‐agnostic screening approaches.

Overall, our findings show that *HFE* p.C282Y homozygote penetrance to clinical disease in a large community cohort was partly explained by the cumulative effects of common genetic variants that influence iron measures in the general population. We showed that polygenic scores for iron and *TF* saturation had the strongest associations with outcomes. We also showed that general population–derived polygenic scores for HH‐related conditions including liver diseases, diabetes, and arthritis also modify penetrance to these respective diseases within p.C282Y homozygotes of men and women. Therefore, including polygenic scores in HH screening and diagnosis may help in estimating prognosis and treatment planning in p.C282Y homozygotes, especially those identified in population screening at younger ages before evidence of clinical endpoints could be present.

### AUTHOR CONTRIBUTIONS

Luke C. Pilling conceived the project, generated data, performed analyses, interpreted results, created the figures, searched literature, and co‐wrote the manuscript. Janice L. Atkins generated data, interpreted results, searched literature, and contributed to the manuscript. David Melzer conceived the project, oversaw interpretation and literature searching, and co‐wrote the manuscript.

### FUNDING INFORMATION

This work was generously funded by an award to David Melzer by the Medical Research Council MR/S009892/1. David Melzer and Luke C. Pilling are supported by the University of Exeter Medical School. Janice L. Atkins is supported by a National Institute of Health and Care Research Advanced Fellowship (NIHR301844). The funders had no input in the study design; in the collection, analysis, and interpretation of data; in the writing of the report; or in the decision to submit the article for publication.

### CONFLICTS OF INTEREST

All authors declare no conflicts of interest.

## Supporting information


**Figures S1‐S3** Transferrin saturation polygenic score associations with HH co‐morbidities in UK Biobank males of European ancestry, stratified by *HFE* genotype
Ferritin polygenic score associations with HH co‐morbidities in UK Biobank males of European ancestry, stratified by *HFE* genotype
Total iron binding capacity polygenic score associations with HH co‐morbidities in UK Biobank males of European ancestry, stratified by *HFE* genotype

**Tables S1‐S8.** xxxx

## Data Availability

The genetic and phenotypic UK Biobank data are available on application to the UK Biobank (www.ukbiobank.ac.uk/register‐apply). The derived data fields used in our analysis will be available via the UK Biobank, searching for application number 14631—we are not able to share these directly.

## References

[1] Adams PC . Epidemiology and diagnostic testing for hemochromatosis and iron overload. Int J Lab Hematol. 2015;37(S1):25–30. 10.1111/ijlh.12347 25976957

[2] Hollerer I , Bachmann A , Muckenthaler MU . Pathophysiological consequences and benefits of HFE mutations: 20 years of research. Haematologica. 2017;102(5):809–17. 10.3324/haematol.2016.160432 28280078 PMC5477599

[3] Sahinbegovic E , Dallos T , Aigner E , Axmann R , Manger B , Englbrecht M , et al. Musculoskeletal disease burden of hereditary hemochromatosis. Arthritis Rheum. 2010;62(12):3792–8. 10.1002/art.27712 20722017

[4] Powell LW , Seckington RC , Deugnier Y . Haemochromatosis. Lancet. 2016;388(10045):706–16. 10.1016/S0140-6736(15)01315-X 26975792

[5] Atkins JL , Pilling LC , Masoli JAH , Kuo CL , Shearman JD , Adams PC , et al. Association of hemochromatosis *HFE* p.C282Y homozygosity with hepatic malignancy. JAMA. 2020;324(20):2048. 10.1001/jama.2020.21566 33231665 PMC7686863

[6] Gallego CJ , Burt A , Sundaresan AS , Ye Z , Shaw C , Crosslin DR , et al. Penetrance of hemochromatosis in HFE genotypes resulting in p.Cys282Tyr and p.[Cys282Tyr];[His63Asp] in the eMERGE Network. Am J Hum Genet. 2015;97(4):512–20. 10.1016/j.ajhg.2015.08.008 26365338 PMC4596892

[7] Rossi E , Jeffrey GP . Clinical penetrance of C282Y homozygous HFE haemochromatosis. Clin Biochem Rev. 2004;25(3):183–90. [Online]. Available from: http://www.ncbi.nlm.nih.gov/pubmed/18458707 18458707 PMC1880832

[8] Radio FC , Majore S , Aurizi C , Sorge F , Biolcati G , Bernabini S , et al. Hereditary hemochromatosis type 1 phenotype modifiers in Italian patients. The controversial role of variants in HAMP, BMP2, FTL and SLC40A1 genes. Blood Cells Mol Dis. 2015;55(1):71–5. 10.1016/j.bcmd.2015.04.001 25976471

[9] de Tayrac M , Roth MP , Jouanolle AM , Coppin H , le Gac G , Piperno A , et al. Genome‐wide association study identifies TF as a significant modifier gene of iron metabolism in HFE hemochromatosis. J Hepatol. 2015;62(3):664–72. 10.1016/j.jhep.2014.10.017 25457201

[10] Pilling LC , Tamosauskaite J , Jones G , Wood AR , Jones L , Kuo CL , et al. Common conditions associated with hereditary haemochromatosis genetic variants: cohort study in UK Biobank. BMJ. 2019;364:k5222. 10.1136/bmj.k5222 30651232 PMC6334179

[11] Girelli D , Busti F , Brissot P , Cabantchik I , Muckenthaler MU , Porto G . Hemochromatosis classification: update and recommendations by the BIOIRON Society. Blood. 2021;139(20):3018–29. 10.1182/blood.2021011338 34601591

[12] Beutler E , Felitti VJ , Koziol JA , Ho NJ , Gelbart T . Penetrance of 845G → A (C282Y) HFE hereditary haemochromatosis mutation in the USA. Lancet. 2002;359(9302):211–8. 10.1016/S0140-6736(02)07447-0 11812557

[13] Fry A , Littlejohns TJ , Sudlow C , Doherty N , Adamska L , Sprosen T , et al. Comparison of sociodemographic and health‐related characteristics of UK Biobank participants with the general population. Am J Epidemiol. 2017;186:1026–34. 10.1093/aje/kwx246 28641372 PMC5860371

[14] Bycroft C , Freeman C , Petkova D , Band G , Elliott LT , Sharp K , et al. The UK Biobank resource with deep phenotyping and genomic data. Nature. 2018;562(7726):203–9. 10.1038/s41586-018-0579-z 30305743 PMC6786975

[15] Thompson WD , Tyrrell J , Borges MC , Beaumont RN , Knight BA , Wood AR , et al. Association of maternal circulating 25(OH)D and calcium with birth weight: a mendelian randomisation analysis. PLoS Med. 2019;16(6):e1002828. 10.1371/journal.pmed.1002828 31211782 PMC6581250

[16] Moksnes MR , Hansen AF , Graham SE , Gagliano Taliun SA , Wu KH , et al. Genome‐wide meta‐analysis of iron status biomarkers and the effect of iron on all‐cause mortality in HUNT. medRxiv. 2021;15:2021.09.20.21262960. 10.1101/2021.09.20.21262960 PMC9203493 35710628

[17] Emdin CA , Haas M , Ajmera V , Simon TG , Homburger J , Neben C , et al. Association of genetic variation with cirrhosis: a multi‐trait genome‐wide association and gene–environment interaction study. Gastroenterology. 2021;160(5):1620–33.e13. 10.1053/j.gastro.2020.12.011 33310085 PMC8035329

[18] Tachmazidou I , Hatzikotoulas K , Southam L , Esparza‐Gordillo J , Haberland V , Zheng J , et al. Identification of new therapeutic targets for osteoarthritis through genome‐wide analyses of UK Biobank data. Nat Genet. 2019;51(2):230–6. 10.1038/s41588-018-0327-1 30664745 PMC6400267

[19] Mahajan A , Taliun D , Thurner M , Robertson NR , Torres JM , Rayner NW , et al. Fine‐mapping type 2 diabetes loci to single‐variant resolution using high‐density imputation and islet‐specific epigenome map. Nat Genet. 2018;50(11):1505–13. 10.1038/s41588-018-0241-6 30297969 PMC6287706

[20] Bowden J , Davey Smith G , Burgess S . Mendelian randomization with invalid instruments: effect estimation and bias detection through Egger regression. Int J Epidemiol. 2015;44(2):512–25. 10.1093/ije/dyv080 26050253 PMC4469799

[21] Bowden J , Spiller W , del Greco FM , Sheehan N , Thompson J , Minelli C , et al. Improving the visualization, interpretation and analysis of two‐sample summary data Mendelian randomization via the radial plot and radial regression. Int J Epidemiol. 2018;47(4):1264–78. 10.1093/ije/dyy101 29961852 PMC6124632

[22] Manichaikul A , Mychaleckyj JC , Rich SS , Daly K , Sale M , Chen WM . Robust relationship inference in genome‐wide association studies. Bioinformatics. 2010;26(22):2867–73. 10.1093/bioinformatics/btq559 20926424 PMC3025716

[23] Anderson GJ , Bardou‐Jacquet E . Revisiting hemochromatosis: genetic vs. phenotypic manifestations. Ann Transl Med. 2021;9(8):731. 10.21037/atm-20-5512 33987429 PMC8106074

[24] Milet J , Déhais V , Bourgain C , Jouanolle AM , Mosser A , Perrin M , et al. Common variants in the BMP2, BMP4, and HJV genes of the hepcidin regulation pathway modulate HFE hemochromatosis penetrance. Am J Hum Genet. 2007;81(4):799–807. 10.1086/520001 17847004 PMC2227929

[25] Sandnes M , Vorland M , Ulvik RJ , Reikvam H . Hfe genotype, ferritin levels and transferrin saturation in patients with suspected hereditary hemochromatosis. Genes (Basel). 2021;12(8):1162. 10.3390/genes12081162 34440336 PMC8394043

[26] Adams PC , Reboussin DM , Barton JC , McLaren C , Eckfeldt JH , McLaren G , et al. Hemochromatosis and iron‐overload screening in a racially diverse population. N Engl J Med. 2005;35217(17):1769–78. 10.1056/NEJMoa041534 15858186

[27] Gurrin LC , Bertalli NA , Dalton GW , Osborne NJ , Constantine CC , McLaren CE , et al. HFE C282Y/H63D compound heterozygotes are at low risk of hemochromatosis‐related morbidity. Hepatology. 2009;50(1):94–101. 10.1002/hep.22972 19554541 PMC3763940

[28] Rishi G , Wallace DF , Subramaniam VN . Hepcidin: regulation of the master iron regulator. Biosci Rep. 2015;35(3):e00192. 10.1042/BSR20150014 26182354 PMC4438303

[29] Béliveau F , Tarkar A , Dion SP , Désilets A , Ghinet MG , Boudreault PL , et al. Discovery and development of TMPRSS6 inhibitors modulating hepcidin levels in human hepatocytes. Cell Chem Biol. 2019;26(11):1559–72.e9. 10.1016/j.chembiol.2019.09.004 31543462

[30] Adams PC . Hepcidin in hemochromatosis: the message or the messenger? Hepatology. 2014;59(3):749–50. 10.1002/hep.26715 23996780

[31] Kohgo Y , Ikuta K , Ohtake T , Torimoto Y , Kato J . Iron overload and cofactors with special reference to alcohol, hepatitis C virus infection and steatosis/insulin resistance. World J Gastroenterol. 2007;13(35):4699–706. 10.3748/wjg.v13.i35.4699 17729391 PMC4611191

[32] McCune CA , Ravine D , Carter K , Jackson HA , Hutton D , Hedderich J , et al. Iron loading and morbidity among relatives of HFE C282Y homozygotes identified either by population genetic testing or presenting as patients. Gut. 2006;55(4):554–62. 10.1136/gut.2005.070342 16174659 PMC1856156

[33] de Graaff B , Neil A , Si L , Yee KC , Sanderson K , Gurrin L , et al. Cost‐effectiveness of different population screening strategies for hereditary haemochromatosis in Australia. Appl Health Econ Health Policy. 2017;15(4):521–34. 10.1007/s40258-016-0297-3 28035629

