# Supplementary material for: Genetic modifiers of penetrance to liver endpoints in HFE hemochromatosis: Associations in a large community cohort
Source: Hepatology. 2022 Jun 17;76(6):1735–45. doi: 10.1002/hep.32575 (PMC9796074; doi:10.1002/hep.32575)

**Genetic modifiers of penetrance to liver endpoints in *HFE* hemochromatosis: associations in a large community cohort**

Luke C. Pilling, Janice L. Atkins, David Melzer

**Supplementary Information**

[Supplementary Figure 1: Transferrin saturation polygenic score associations with HH co-morbidities in UK Biobank males of European ancestry, stratified by *HFE* genotype 2](#_Toc102653440)

[Supplementary Figure 2: Ferritin polygenic score associations with HH co-morbidities in UK Biobank males of European ancestry, stratified by *HFE* genotype 3](#_Toc102653441)

[Supplementary Figure 3: Total iron binding capacity polygenic score associations with HH co-morbidities in UK Biobank males of European ancestry, stratified by *HFE* genotype 4](#_Toc102653442)

# Supplementary Figure 1: Transferrin saturation polygenic score associations with HH co-morbidities in UK Biobank males of European ancestry, stratified by *HFE* genotype


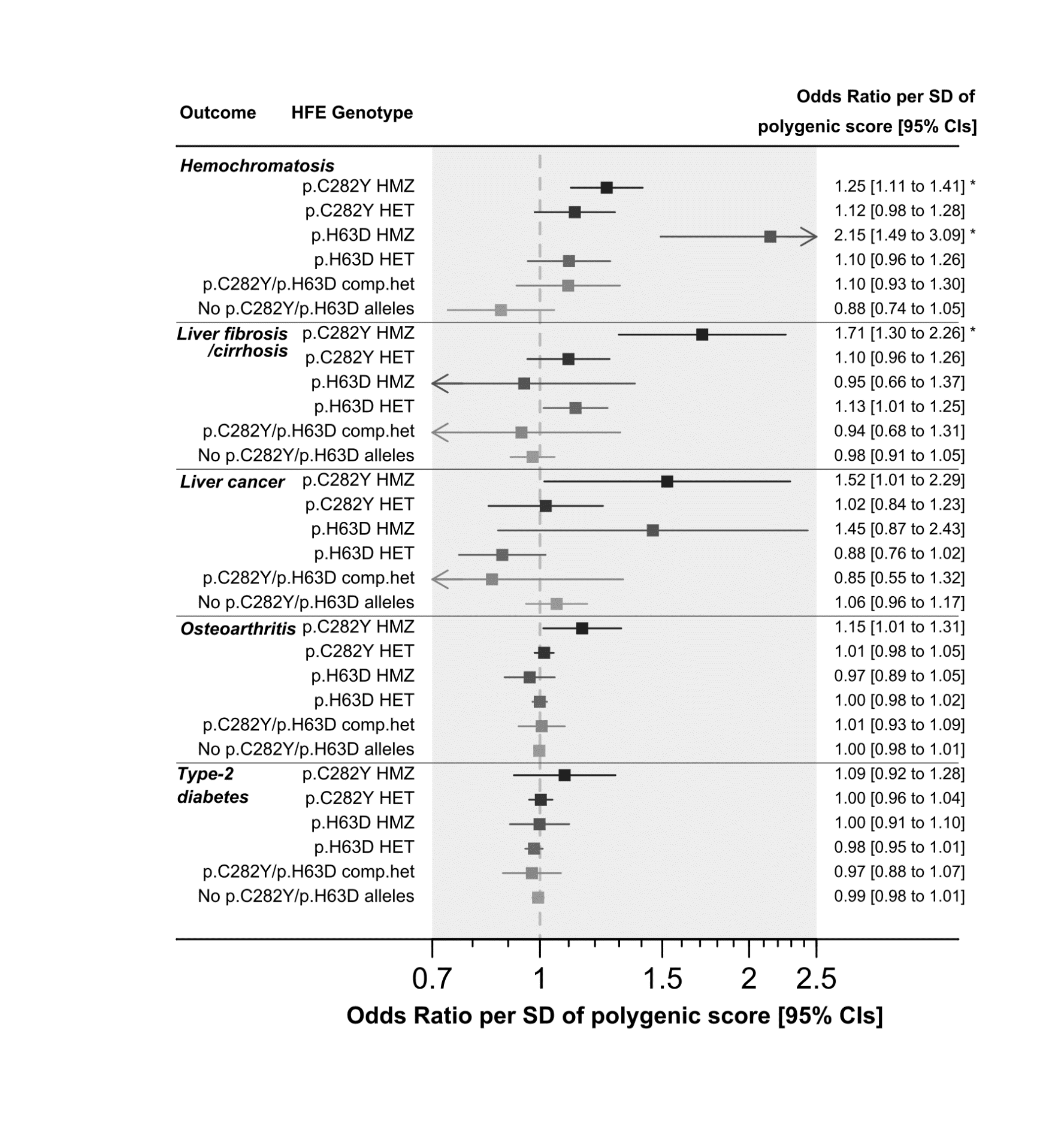


# Supplementary Figure 2: Ferritin polygenic score associations with HH co-morbidities in UK Biobank males of European ancestry, stratified by *HFE* genotype


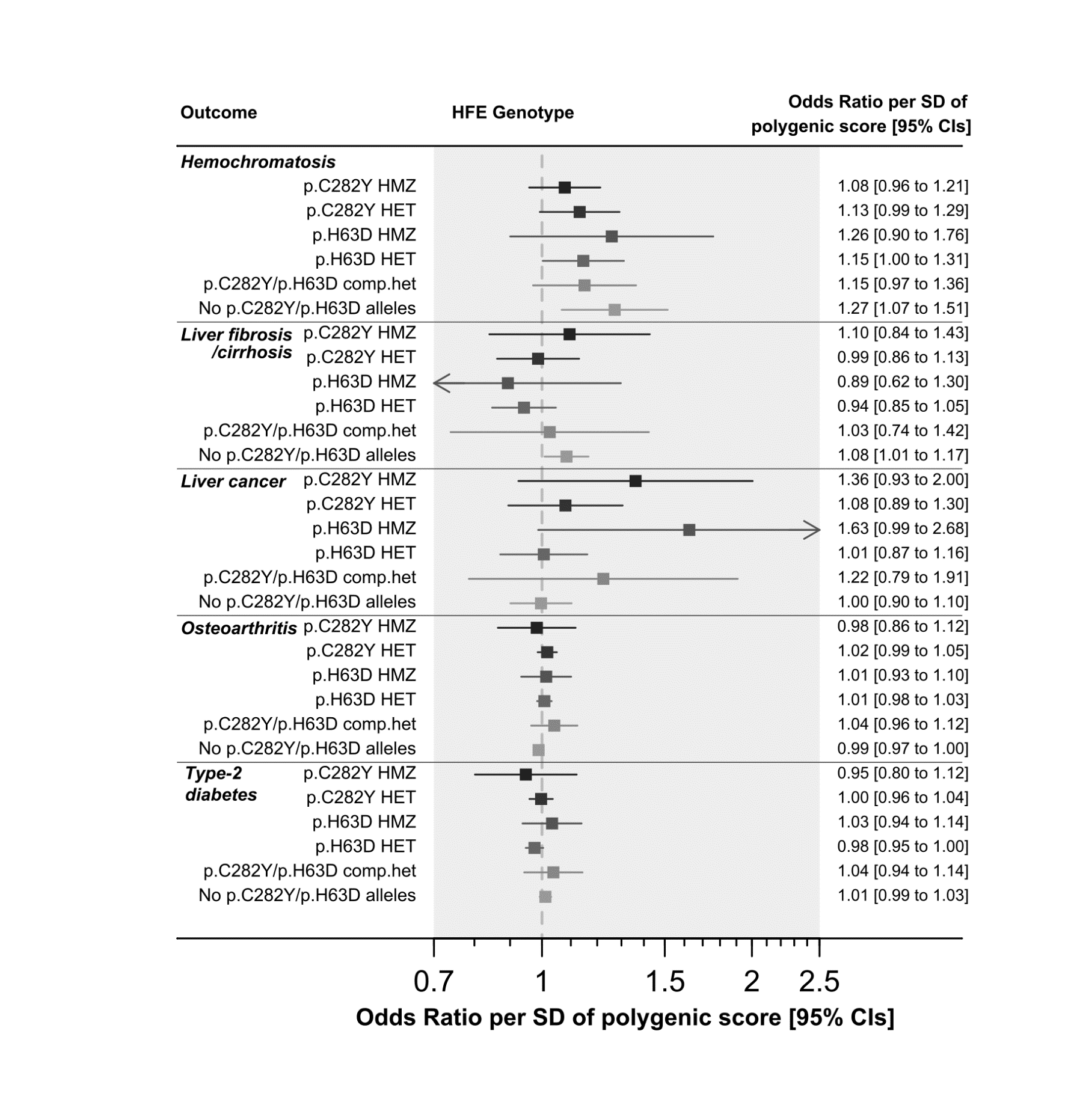


# Supplementary Figure 3: Total iron binding capacity polygenic score associations with HH co-morbidities in UK Biobank males of European ancestry, stratified by *HFE* genotype

Note: TIBC is coded in the negative direction (as per the HUNT study) therefore an OR<1 refers to greater TIBC *increasing* the Odds Ratio.


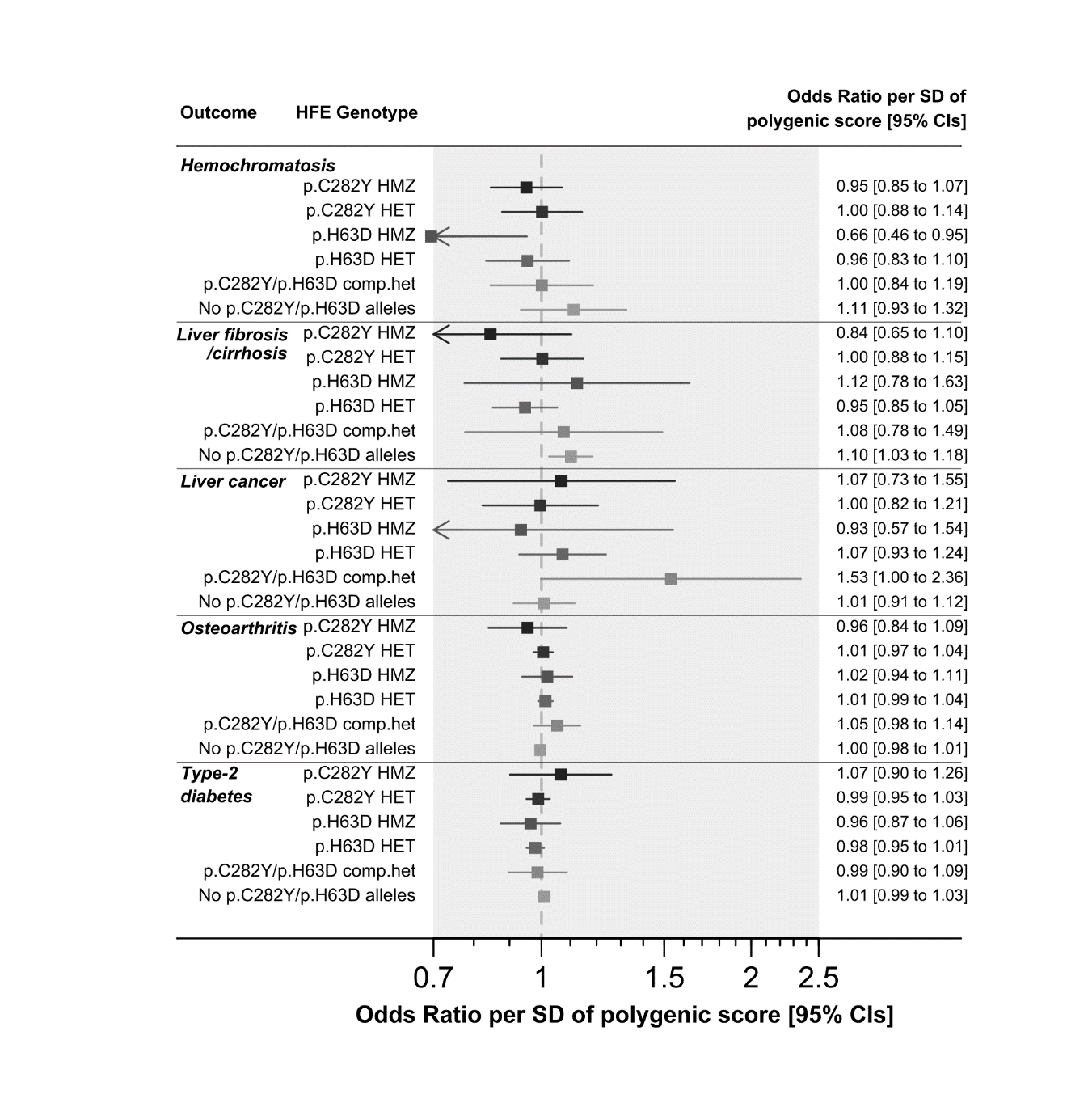

Supplement: Supplementary file 1 — Figures S1‐S3 Transferrin saturation polygenic score associations with HH co‐morbidities in UK Biobank males of European ancestry, stratified by HFE genotype Ferritin polygenic score associations with HH co‐morbidities in UK Biobank males of European ancestry, stratified by HFE genotype Total iron binding capacity polygenic score associations with HH co‐morbidities in UK Biobank males of European ancestry, stratified by HFE genotype [file HEP-76-1735-s002.docx]
